# Cross-Cultural Adaptation of the Brazilian Portuguese-Translated Version of the Mini Sarcopenia Risk Assessment (MSRA) Questionnaire in Cancer Patients

**DOI:** 10.3390/clinpract11020054

**Published:** 2021-06-16

**Authors:** Lays S. Ribeiro, Bárbarah G. de A. Souza, Juliana B. de Lima, Gustavo D. Pimentel

**Affiliations:** 1 Hospital das Clínicas de Goiás, Universidade Federal de Goiás, Goiânia 74605-080, Brazil; nutri.laysribeiro@gmail.com (L.S.R.); barbarahgregorio@gmail.com (B.G.d.A.S.); julianablima.nutri@gmail.com (J.B.d.L.); 2 Laboratório de Investigação em Nutrição Clínica e Esportiva (Labince), Faculdade de Nu-trição, Universidade Federal de Goiás, Goiânia 74605-080, Brazil

**Keywords:** nutritional assessment, sarcopenia, oncology, hospital

## Abstract

Background and Aims: This study sought (i) to adapt cross-culturally the Brazilian Portuguese-translated version of the MSRA questionnaire, (ii) to estimate the prevalence of risk of sarcopenia, and (iii) to correlate the MSRA Portuguese version with CC in hospitalized cancer patients. Methods: This was a cross-sectional study developed at the hospital in the year 2018. After being translated and adapted to the Portuguese language, the questionnaire was applied and associated with the measurement of calf circumference (CC) to verify its validity. Results: Of the 45 patients, 71.1% presented significant or severe body weight loss, and 55.5% indicated muscle mass loss (CC < 31 cm). From the MSRA of seven and five items, 91.1 and 84.5% of the patients, respectively, presented risk for sarcopenia. Among those at risk for sarcopenia, more than 60% were aged <70 years, >80% were hospitalized in the last year, more than 40% could not walk > 1000 m, >40% did not eat regularly, and >80% lost >2 kg in the last year. CC was correlated with MSRA of five items (r = 0.46, *p* = 0.001) and seven items (r = 0.53, *p* = 0.0002). Furthermore, both versions of the MSRA (seven and five items) presented strong agreement and high reliability. Conclusions: The MSRA full and short version was adapted for a Brazilian Portuguese-translated version and showed strong agreement and high reliability to identify the risk for sarcopenia in hospitalized cancer patients. Therefore, this questionnaire can be used as part of nutritional assessment protocols in order to enable early screening of the risk for sarcopenia

## 1. Introduction

According to the European Working Group on Sarcopenia in Older People (EWGSOP2), sarcopenia is a skeletal muscle loss associated with loss of muscle function [1]. A Brazilian study identified presence of sarcopenia in ~44% of cancer patients and of these, 86% had increased risk of death [2].

In clinical practice, magnetic resonance imaging (MRI) and computed tomography (CT) are recommended as the gold standard for quantitative non-invasive assessment of muscle mass. Dual-energy X-ray absorptiometry, bioimpedance analysis, or anthropometric measures can also be used [1]. Nevertheless, this equipment is quite expensive and are not usually accessible, especially in public health services. This condition leads to under-diagnosis of sarcopenia. Thus, EWGSOP2 [1] recommends the use of a validated tool for screening the risk of sarcopenia.

Although the Strength, Assistance with walking, Rise from a chair, Climb stairs, and falls questionnaire (SARC-F) [3] has been validated for a Portuguese version, it is better correlated with sarcopenia when the SARC-F is evaluated with calf circumference (CC) [4]. Likewise, Mini Sarcopenia Risk Assessment (MSRA) is a sarcopenia screening tool developed for attending health centers in the city of Verona, Italy. This questionnaire has two versions (MSRA-7, full version with seven items and MSRA-5, short version with five items) which are sensitive for the identification of the risk of sarcopenia [5]. However, this instrument needed to be validated for a Brazilian Portuguese version and to confirm its correlation with CC measurement in hospitalized cancer patients.

A recent study validated MSRA for a Chinese population and found a better sensitivity for MSRA-5 and better specificity for SARC-F for screening sarcopenia, suggesting that MSRA use in different populations requires further investigation [6].

Considering that in hospitals it is essential to use simple and sensitive instruments, since gold-standard tools for the evaluation of sarcopenia are costly [7], this pilot study sought (i) to adapt cross-culturally the Brazilian Portuguese-translated version of the MSRA questionnaire, (ii) to estimate the prevalence of risk of sarcopenia, and (iii) to correlate the MSRA Portuguese version with CC in hospitalized cancer patients.

## 2. Methods

### 2.1. Study Design

This cross-sectional study was developed at the Hospital das Clínicas de Goiás, Goiás, Brazil.

### 2.2. Patients and Ethical Procedures

The study sample consisted of hospitalized cancer patients aged 52.1 ± 16.0 y, recruited at the Medical, Surgical, Tropical, and Emergency Clinics, and undergoing chemotherapy at the Hospital das Clínicas. Patients in the Intensive Care Unit, pregnant women, and those with lower limb edema were excluded.

The study was approved by the Research Ethics Committee at the Hospital das Clínicas (approval No.: 2.674.012). All the participants signed a free and informed consent form, informing them about the research and the risks involved.

### 2.3. Procedures for Cross-Cultural Adaptation of the MRSA

MSRA is a questionnaire used for screening the sarcopenia that was elaborated to use in Italian subjects and has been validated in Chinese, Polish and Thai languages [5,6,8,9].

The transcultural translation and adaptation of the questionnaire was undertaken based on the procedures recommended by Beaton et al. [10], revised by Muñiz et al. [11].

Before translation of the original MSRA questionnaire (Supplementary Table S1) [5], authorization from the associated authors was obtained via e-mail. In addition, a committee of experts on this issue was designed to discuss concepts of the MSRA, considering the particularities of the cancer patients and the aims of the questionnaire.

All translators of the study were unaware of the test to be adapted. Two translators were selected for each step (translation and back translation). In the first phase, one of the chosen translators did not specialize in the outcome investigated (sarcopenia in onco–hematological patients) by the test, in order to preserve the representativeness of the popular use of the target language.

In the 2nd phase, after synthesis of the Portuguese translations, its applicability was verified through paraphrase, a strategy in which the interviewer asks the question and asks the interviewee to repeat it immediately. No operational difficulties were noticed, therefore, no further adjustments of the test by the committee were necessary and the Portuguese synthesis was sent for back translation.

The back translation (3rd phase) was performed by an English native speaker who compared the synthesized version with the original. Then, the elaboration of the final Portuguese version (4th phase) was organized.

Regarding cross-cultural adaptation, only two modifications were required. We removed the term “ragout” (ragu, in Portuguese) and “ham” (presunto, in Portuguese) both from item 6 of the questionnaire, since it is not a dish typically consumed by Brazilians, unlike Italy, where the MSRA was originally developed. Additionally, ham is an unhealthy food for cancer patients to eat, since processed meat can enhance the risk of cancer.

### 2.4. Data Collection and Risk Classification of Sarcopenia

Age, sex, cancer diagnosis, and percentage and time of body weight loss were collected during medical and nutritional consultation. The application of the questionnaire and the data collection were carried out from June to December 2018, by trained nutritionists with experience in hospital clinical nutrition, using the instrument obtained from the Portuguese translation, titled “Mini Questionário de Avaliação de Risco de Sarcopenia” (Supplementary Table S2).

The MSRA consists of 7 items related to the general assessment (questions related to age, physical activity level, hospitalizations, and weight loss) and dietary assessment (number of meals per day, consumption of dairy products and proteins), selected based on a review of the literature on risk factors for decreased muscle mass and strength loss [5].

In addition to the 7-item version, the questionnaire can also be applied in a reduced 5-item version [5]. In the present study, we adopted the same risk classification for sarcopenia as the original study [5]. Thus, for the 7-item MSRA, scores ≤30 indicate risk of sarcopenia and, for the 5-item MSRA, scores ≤45 indicate risk of sarcopenia.

In addition to the interview using MSRA, CC was measured only in the non-dominant leg with the patient in sitting position. The objective is to correlate the instrument studied and another measure related to the same outcome. CC values <31 cm indicate loss of lean body mass and probable association with sarcopenia [12].

### 2.5. Statistical Analyses

Data normality was tested using the Kolmogorov–Smirnov test. Values were presented as mean ± standard deviation. Estimates of the prevalence of sarcopenia risk were assessed by percentage. Cronbach’s alpha [13] measures the alpha coefficient which estimates correlation between two measurements. It evaluated internal consistency (reliability) between items of the MRSA questionnaire, and for classification, its represented the maximum value as 1 (high reliability), and minimum as 0 (low reliability). For these statistical analyses the R software version 3.2.1 was used, using the electronic address (http://langtest.jp/shiny/rel/, accessed on 9 June 2021).

The Bland–Altman test was applied to evaluate the agreement between MSRA questionnaire items (MSRA-7, full version and MSRA-5, short version). The Bland–Altman test and kappa (*κ*) coefficient were computed using the MedCalc^®^ software, Ostend, Belgium. The quadratic weighted kappa test was used to assess the agreement between the two questionnaires using the numerical variables of the MSRA scores of both MSRA-7 (full version) and MSRA-5 (short version), where *κ* ≤ 0.20 = poor agreement; 0.21 ≤ *κ* ≤ 0.40 = fair; 0.41 ≤ *κ* ≤ 0.60 = moderate; 0.61 ≤ *κ* ≤ 0.80 = good; and 0.81 ≤ *κ* ≤ 1.00 = very good. Pearson´s test was used to assess the correlation between MRSA with 5 and 7 items and with CC. Chi-square or Fisher test were used to evaluate the differences between patients with risk versus no risk for sarcopenia and possible differences between MSRA-5 and MSRA-7. Values of *p* < 0.05 were considered as significant.

## 3. Results

The final Portuguese version of the MSRA is presented in Supplementary Table S2. The estimated prevalence of the risk of sarcopenia among patients using the MSRA-7 was 91.1%, and 84.5% using the MRSA with five items, respectively.

Of the 45 patients included in the study, 46.7% (*n* = 21) were males, and 53.3% (*n* = 24) were females (Table 1). The diagnoses most prevalent were lymphomas and leukemias, and intestinal tumors. Approximately 71.1% of the patients presented significant body weight loss, of which, 20% (*n* = 9) presented a significant loss, and 51% (*n* = 23) showed severe body weight loss; 55.5% (*n* = 25) indicated loss of muscle mass (CC < 31 cm), and 91 (*n* = 41) and 84.5% (*n* = 38) presented risk of sarcopenia using the seven- and five-item MSRA, respectively (Table 1).

Of the patients classified with risk of sarcopenia by the seven-item MSRA, 68% were aged < 70 years, 80% were hospitalized in the last year, 41% could not walk > 1000 m, 44% did not consume full meals regularly, > 441.5% did not consume protein-based foods regularly, and 80% had lost > 2 kg in the last year. The five-item MSRA presented similar results; 66% were aged < 70 years old, 84% were hospitalized in the last year, 45% could not walk > 1000 m, 47% did not consume full meals regularly, and 81.5% had lost > 2 kg in the last year (Table 2). No difference between the seven- and five-item MSRA versions was found for age, physical activity level, and body weight loss (Table 2). Additionally, the MSRA-5 questionnaire identified more patients as non-sarcopenic with at least one or no hospitalizations in the last year compared with the MSRA-7 questionnaire (*p* = 0.006). Moreover, the MSRA-5 questionnaire also had more non-sarcopenic patients who ate at least three meals per day regularly than MSRA-7 (*p* = 0.037) (Table 2).

**Figure 1:**
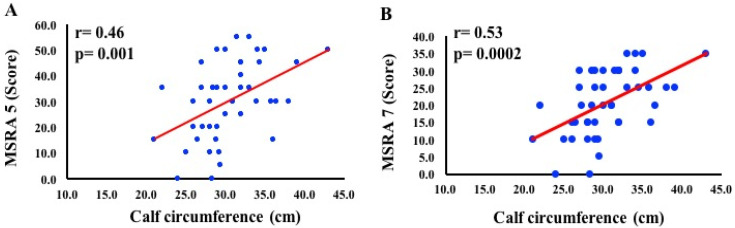
Correlation between calf circumference and Mini Sarcopenia Risk Assessment Questionnaire (MSRA) of 5 (short version, **A**) and 7 (full version, **B**) items. *p* < 0.05 was considered as significant.

**Figure 2:**
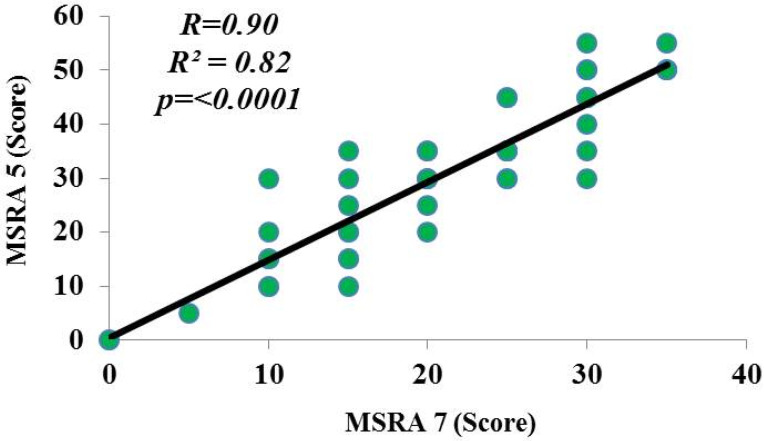
Correlation coefficient between MSRA-7 (full version) and -5 (short version) items. MSRA: Mini Sarcopenia Risk Assessment questionnaire. *p* < 0.05 was considered as significant.

**Figure 3:**
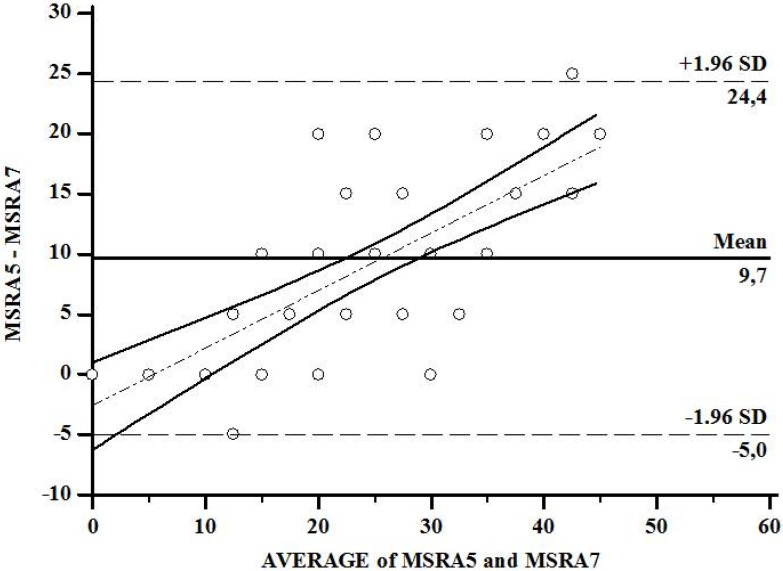
Bland–Altman coefficient analysis between MSRA-7 (full version) and -5 (short version) items. MSRA: Mini Sarcopenia Risk Assessment questionnaire.

In addition, CC was correlated with the MSRA of five items (r = 0.46, *p* = 0.001) and seven items (r = 0.53, *p* = 0.0002) (Figure 1). Besides, a positive correlation between the seven- and five-item MSRA scores (*r*^2^ = 0.82, *r* = 0.90, *p* < 0.0001) (Figure 2), and a strong concordance by both the *κ* index (0.622) and standard error (0.102) (Figure S1) with the Bland–Altman coefficient, with a mean difference of 9.7, and a concordance limit of 7.35 (Figure 3), were found. In addition, the Cronbach’s alpha coefficient was 0.90 (95% CI 0.55–1.24), indicating an excellent internal consistency (high reliability) between the seven- and five-item MSRA versions.

## 4. Discussion

The main finding of the present study was that both versions of the MSRA (seven items—full version and five items—short version) are reliable since they present high agreement and correlation with each other. Therefore, additional studies may apply this low-cost tool to facilitate the screening of sarcopenia in hospitalized patients. Additionally, to our knowledge, this study is the first to apply the MSRA to cancer patients. The original version of the MSRA applied to the elderly patients assisted at outpatient services in the city of Verona, Italy [5]. Our study translated, adapted according to the Brazilian culture, both versions (seven and five items) of the screening risk of sarcopenia questionnaire for hospitalized patients.

Since sarcopenia is considered a type of muscular dystrophy (ICD-10-CM), easy-to-use questionnaires are used in health services to screen sarcopenia [1]. However, they still lack uniform criteria for the assessing of sarcopenia [6].

In the present study, those with higher body weight loss (71%) also presented CC < 31 cm (55.5%). Additionally, CC was correlated with the seven- and five-item MSRA, indicating a loss of lean body mass and increased risk for sarcopenia. These data agree with Barbosa-Silva et al. [4], who observed that SARC-F, an easy-to-use questionnaire when associated with CC, significantly improved the screening ability for sarcopenia. Therefore, these results point to the physiological mechanism of involuntary reduction in body weight in cases of increased metabolic demand, such as in cancers, where there is accelerated catabolism with reduction in fat deposits and lean body mass, leading patients to morbidity and mortality.

Among those considered at risk for sarcopenia in both questionnaires (seven- and five-item MSRA), >65% are aged <70 years, corroborating with other studies [1,14] that demonstrated the importance of screening the risk of sarcopenia.

Indeed, sarcopenia may increase the risk of hospitalization [1]. In the present study, of the patients classified as having no risk for sarcopenia, >70% were not hospitalized in the last year. Conversely, in patients at risk for sarcopenia, >80% reported having been hospitalized in the last year, which contributes to the increased cost of health treatments, worsening of prognosis, and mortality. In addition, inability to walk >1000 m was reported by >40% of patients screened with risk for sarcopenia, a striking factor in the quality of life. Bye et al. [15] found that cancer patients with decreased muscle mass presented worse physical function and autonomy, and difficulty in performing daily activities.

In the present study, 50% of the patients had a deficit in maintaining food regularity, and 30% reported not regularly consuming food protein sources. The reduction in caloric and protein intake, associated with the metabolic demand of tumors, leads to malnutrition. Likewise, we reinforce the importance of the complete and regular nutritional evaluation of cancer patients from their hospital admission, with adequate nutritional counselling.

Approximately 50% of cancer patients present body weight loss and malnutrition. The nutritional status deficit is associated with reduced response to treatment and quality of life [16,17]. In our study, >80% of patients with risk of sarcopenia lost body weight in the last year, and more than 50% had severe weight loss.

Recent Brazilian studies have already shown a higher prevalence of sarcopenia in cancer patients. A study developed in Campinas City involving colorectal cancer patients found that 44% had some degree of sarcopenia [2]. In the present study, 91 (*n* = 41) and 84.5% (*n* = 38) of patients presented risk for sarcopenia, according to the MSRA-7 and -5, respectively. The high percentage seems to be associated with the disease condition, number and length of hospitalization, cancer treatments, and inflammatory profile, among other factors.

Additionally, Chinese researchers also validated the MSRA for chronic diseases in older patients. The prevalence of the risk of sarcopenia was 33.6% in the seven-items and 38% in the five-items version, respectively [6]. Therefore, these data seem to strengthen the importance of early screening for sarcopenia in patients with chronic diseases.

The present study has some limitations: (1) The evaluation of the muscle mass indicator was performed only by the measurement of the CC. Although the EWGSOP [1] recommends the use of magnetic resonance imaging and computed tomography as the gold standard for the evaluation of muscle mass, these methods are expensive and not accessible to the majority of public hospitals; (2) Although there is association between CC and MSRA, these methods must be used only for screening the risk for sarcopenia, and are not able to diagnose the presence or absence of sarcopenia; (3) Studies using MSRA were performed with out-of-hospital elderly patients. We studied the application of this questionnaire in adults and older cancer patients. Therefore, it is expected that hospitalized patients may be affected earlier by sarcopenia than outpatients; thus further studies are warranted to investigate older cancer patients; (4) The small sample size requires further validation and suggests caution in interpreting the data, and further studies are being conducted to investigate the inference of these findings using gold standard techniques; and (5) Inclusion of a single center diminishes the potential generalizability of data; thus, further studies are warranted to include more research centers. On the other hand, some positive points must be highlighted due to advantages for clinical practice: (1) it is an adaptation of a Brazilian Portuguese-translated version questionnaire for screening of sarcopenia risk in cancer patients, rather than Italian, Thai [8], and Polish [9]; (2) we suggest that associated with other nutritional assessment tools, the questionnaire will be useful for clinical practice in hospitals; and (3) the questionnaire is an easy-to use tool which will allow a fast screening for sarcopenia, especially in local hospitals with under-diagnosis of sarcopenia.

## 5. Conclusions

This pilot study showed that the MSRA questionnaire was adapted for a Brazilian Portuguese-translated version, and there was high reliability and strong agreement between the two versions (MSRA-7 and -5, full and short version, respectively) when applied to hospitalized cancer patients. Therefore, this questionnaire can be used as part of nutritional assessment protocols, with the objective of screening for the risk of sarcopenia. Furthermore, longitudinal studies with a larger sample size, including different populations, will aid the clarification of the best use of this questionnaire for cancer patients.

## Figures and Tables

**Table 1:** Social, clinical, and anthropometric characteristics of patients.

Variables	N (%)	Mean ± SD
Sex Male Female	21 (46.7) 24 (53.3)	
Age (years)		52.1 ± 16.0
Cancer type Oncohematological Gastrointestinal tract Others	22 (48.9) 19 (42.2) 4 (8.9)	
Body weight loss (%)		12 ± 7.2
Body weight loss time (weeks)		16.97 ± 14.35
Body weight loss Non-significant Significant Severe	13 (28.9) 9 (20.0) 23 (51.1)	
Calf circumference (cm)		30.4 ± 4.2
Calf circumference No muscle mass loss (CC >31 cm) With muscle mass loss (CC <31 cm)	20 (44.5) 25 (55.5)	
MSRA 7 items—full version (score) No risk of sarcopenia With risk of sarcopenia	4 (8.9) 41 (91.1)	20.7 ± 9.2 35.0 ± 0.0 18.4 ± 8.5
MSRA 5 items—short version (score) No risk of sarcopenia With risk of sarcopenia	7 (15.5) 38 (84.5)	51.4 ± 2.4 26.5 ± 12.6

SD: standard deviation; CC: calf circumference.

**Table 2:** Number of patients without risk and at risk of sarcopenia according to MSRA of 7 and 5 items.

Questions	MSRA 7 Items			MSRA 5 Items			*p* Interaction
	NS	S	*p*	NS	S	*p*	
	(*n* = 4)	(*n* = 41)		(*n* = 7)	(*n* = 38)		
	N	N		N	N		
1. How old are you?			0.307			0.089	0.161
≥70 years old	0	13		0	13		
<70 years old	4	28		7	25		
2. Were you hospitalized in the last year?			0.035 *			0.003 *	0.006 *
Yes, and more than one hospitalization	0	21		0	21		
Yes, one hospitalization	1	12		2	11		
No	3	8		5	6		
3. What is your activity level?			0.281			0.034 *	0.052
I’m able to walk less than 1000 meters	0	17		0	17		
I’m able to walk more than 1000 meters	4	24		7	21		
4. Do you eat 3 meals per day regularly?			0.138			0.031 *	0.037 *
No, up to twice per week I skip a meal (for example I skip breakfast or I have only milky coffee or soup for dinner)	0	18		0	18		
Yes	4	23		7	20		
5. Do you consume any of the following?			0.138			-	-
Milk or dairy products (yogurt, cheese), but not every day	0	18		-	-		
Milk or dairy products (yogurt, cheese) at least once per day	4	23					
6. Do you consume any of the following?			0.280			-	-
Poultry, meat, fish, eggs, legumes, ragout, or ham, but not every day	0	16		-	-		
Poultry, meat, fish, eggs, legumes, ragout, or ham at least once per day	4	25					
7. Did you lose weight in the last year?			1.000			0.613	0.929
>2 kg	3	33		5	31		
≤2 kg	1	8		2	7		

MSRA: Mini Sarcopenia Risk Assessment Questionnaire; NS: non-sarcopenia; S: sarcopenia. * Chi-square or Fisher test with *p* < 0.05.

## Supplementary Materials

The following are available online at https://www.mdpi.com/article/10.3390/clinpract11020054/s1, Table S1: Mini Sarcopenia Risk Assessment (MSRA) questionnaire. Table S2: Portuguese version of the Mini Questionário de Avaliação de Risco de Sarcopenia (MARS). Figure S1: Agreement analysis (Kappa index) between MSRA 7 and 5 items. MSRA: Mini Sarcopenia Risk Assessment Questionnaire.
