# Supplementary material for: Cross-Cultural Adaptation of the Brazilian Portuguese-Translated Version of the Mini Sarcopenia Risk Assessment (MSRA) Questionnaire in Cancer Patients
Source: Clin Pract. 2021 Jun 16;11(2):395–403. doi: 10.3390/clinpract11020054 (PMC8293221; doi:10.3390/clinpract11020054)
Supplement: Supplementary file 1 [file clinpract-11-00054-s001.zip › clinpract-1157541-supplementary.pdf]

Supplementary data

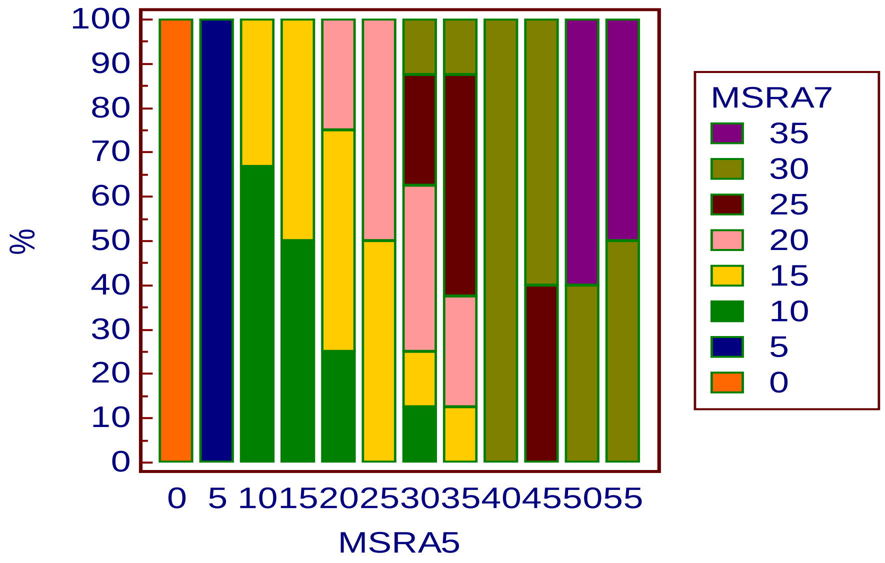

S1. Agreement analysis (Kappa index) between MSRA 7 and 5 items. MSRA: Mini Sarcopenia Risk Assessment Questionnaire.

S Table 1. Mini Sarcopenia Risk Assessment (MSRA) questionnaire.

**Questions**		**7 items**	**5 items**
1-How old are you? Score Score	≥70 years	0	0
	<70 years	5	5
2-Were you hospitalized in the last year?	Yes, and more than one hospitalization	0	0
	Yes, one hospitalization	5	10
	No	10	15
3-What is your activity level?	I'm able to walk less than 1000 meters	0	0
	I'm able to walk more than 1000 meters	5	15
4-Do you eat 3 meals per day regularly?	No, up to twice per week I skip a meal (for example I skip breakfast or I have only milky coffee or soup for dinner)	0	0
	Yes	5	15
5-Do you consume any of the following?	Milk or dairy products (yogurt, cheese), but not every day	0	-
	Milk or dairy products (yogurt, cheese) at least once per day	5	-

6-Do you consume any of the following?	Poultry, meat, fish, eggs, legumes, ragout or ham, but not every day	0	-
	Poultry, meat, fish, eggs, legumes, ragout or ham at least once per day	5	-
7-Did you lose weight in the last year?	>2 kg	0	0
	≤2 kg	5	10

S Table 2. Portuguese version of the Mini Questionário de Avaliação de Risco de Sarcopenia (MARS).

Questões		7 itens	5 itens
1. Quantos anos você tem?	≥ 70 anos de idade	0	0
	< 70 anos de idade	5	5
2. Você foi hospitalizado no ultimo ano?	Sim, e mais de uma hospitalização.	0	0
	Sim, uma hospitalização.	5	10
	Não.	10	15
3. Qual o seu nível de atividade?	Eu consigo andar menos de 1000 metros.	0	0
	Eu consigo andar mais de 1000 metros.	5	15
4. Você consome três refeições por dia regularmente?	Não, até duas vezes por semana eu pulo uma refeição (por exemplo, eu pulo o café da manhã ou tomo somente leite com café ou sopa no jantar).	0	0
	Sim.	5	15
5. Você consome algum dos seguintes alimentos?	Leite ou derivados do leite (iogurte, queijo), mas não todos os dias.	0	-
	Leite ou derivados do leite (iogurte, queijo) pelo menos uma vez ao dia.	5	-
6. Você consome algum dos seguintes alimentos?	Aves, carne, peixe, ovos, leguminosas, mas não todos os dias.	0	-
	Aves, carne, peixe, ovos ou leguminosas, pelo menos uma vez ao dia.	5	-
7. Você perdeu peso no ultimo ano?	> 2 kg	0	0
	≤ 2 kg	5	10
